# Iron phthalocyanine with coordination induced electronic localization to boost oxygen reduction reaction

**DOI:** 10.1038/s41467-020-18062-y

**Published:** 2020-08-20

**Authors:** Kejun Chen, Kang Liu, Pengda An, Huangjingwei Li, Yiyang Lin, Junhua Hu, Chuankun Jia, Junwei Fu, Hongmei Li, Hui Liu, Zhang Lin, Wenzhang Li, Jiahang Li, Ying-Rui Lu, Ting-Shan Chan, Ning Zhang, Min Liu

**Affiliations:** 1 State Key Laboratory of Powder Metallurgy, School of Physical and Electronics, Central South University, Changsha, 410083 China; 2 School of Materials Science and Engineering, Central South University, Changsha, 410083 China; 3 School of Materials Science and Engineering, Zhengzhou University, Zhengzhou, 450002 China; 4 College of Materials Science and Engineering, Changsha University of Science & Technology, Changsha, 410114 China; 5 School of Metallurgy and Environment, Central South University, Changsha, 410083 China; 6 School of Chemistry and Chemical Engineering, Central South University, Changsha, 410083 China; 7 Changjun High School of Changsha, Changsha, 410002 China; 8 National Synchrotron Radiation Research Center, 300 Hsinchu, Taiwan

**Keywords:** Electrocatalysis, Renewable energy, Electrocatalysis

## Abstract

Iron phthalocyanine (FePc) is a promising non-precious catalyst for the oxygen reduction reaction (ORR). Unfortunately, FePc with plane-symmetric FeN_4_ site usually exhibits an unsatisfactory ORR activity due to its poor O_2_ adsorption and activation. Here, we report an axial Fe–O coordination induced electronic localization strategy to improve its O_2_ adsorption, activation and thus the ORR performance. Theoretical calculations indicate that the Fe–O coordination evokes the electronic localization among the axial direction of O–FeN_4_ sites to enhance O_2_ adsorption and activation. To realize this speculation, FePc is coordinated with an oxidized carbon. Synchrotron X-ray absorption and Mössbauer spectra validate Fe–O coordination between FePc and carbon. The obtained catalyst exhibits fast kinetics for O_2_ adsorption and activation with an ultralow Tafel slope of 27.5 mV dec^−1^ and a remarkable half-wave potential of 0.90 V. This work offers a new strategy to regulate catalytic sites for better performance.

## Abstract (web summary)

Iron phthalocyanine with a 2D structure and symmetric electron distribution around Fe-N_4_ active sites is not optimal for O_2_ adsorption and activation. Here, the authors report an axial Fe–O coordination induced electronic localization strategy to enhance oxygen reduction reaction performance.

## Introduction

Since the oxygen reduction reaction (ORR) directly determines the energy efficiency of fuel cells and metal–air batteries, catalytic activation of oxygen (O_2_) to accelerate the kinetics of ORR is crucial for these devices^1–6^. Though platinum (Pt)-based catalysts exhibit excellent O_2_ adsorption and activation abilities in ORR, the high price and low reserve sternly restrict their large-scale applications^7–10^. Exploring non-Pt ORR catalysts with high efficiency is imperative for further development of fuel cells and metal–air batteries^11–13^. Among the reported non-Pt ORR catalysts, iron phthalocyanine (FePc) molecular catalyst has aroused much attentions due to its special FeN_4_ active site and low reaction energy barrier during ORR processes^14–16^. However, FePc possesses a typical two dimensional and plane symmetric structure, which leads to the symmetric electron distribution in the well-defined FeN_4_-active sites and is not conducive to the O_2_ adsorption and activation^17,18^. Therefore, breaking the symmetry of electronic density would be an effective strategy to enhance the O_2_ adsorption/activation and then greatly improve the ORR activity of the FePc catalyst.

From the molecular structure, FePc with a tetracoordinate planar FeN_4_ center offers extra coordination sites in the axial direction^19,20^, suggesting the symmetric electronic density can be modulated by suitable axial coordination^20–23^. Generally, the organic ligands with rich electron-donating functional groups, including oxygenic, nitrogenous, and sulfurous species, can be easily employed to coordinate with FePc^24–26^. However, organic ligands are not favored for electrocatalysis due to their poor conductivity. Modifying the surface of conductive carbon materials with oxygenic groups for stronger electron donation to FePc provides an alternative way to overcome the problem of conductivity, and realize axial coordination of O–FeN_4_ sites^17,26,27^. The axial coordination of O–FeN_4_ can break the electronic distribution symmetry of Fe, leading to better oxygen adsorption and activation abilities, and superior ORR activity than those of the FePc catalyst with symmetric FeN_4_ sites.

In this work, we design a composite catalyst (FeAB–O) by coordination of the FePc molecule with oxygen-containing groups on an O_2_ plasma-treated acetylene black (AB–O) matrix to achieve efficient O_2_ adsorption and ORR. Theoretical calculations show that the axial O coordination (O–FeN_4_) sites greatly break the electronic distribution symmetry of Fe and lead to electron localization on O. The obvious electronic localization on O–FeN_4_ sites is beneficial for the axial O_2_ adsorption and activation. X-ray adsorption experiments and the O_2_ adsorption/desorption tests confirm the axial O coordination and outstanding O_2_ adsorption capacity of the FeAB–O catalyst, respectively. The ORR performance measurements show that the optimized FeAB–O catalyst has an ultralow Tafel slope of 27.5 mV dec^−1^ and a superior half-wave potential of 0.90 V vs. reversible hydrogen electrode (RHE), which is 30 and 50 mV higher than FePc supported onto acetylene black (AB) without axial O coordination (FePc/AB) and Pt/C, respectively. This work opens a new avenue to improve the ORR performance of metal phthalocyanine catalysts, and inspires electronic localization of active sites for regulating catalytic reaction activity.

## Results

### Theoretical calculations

**Fig. 1:**
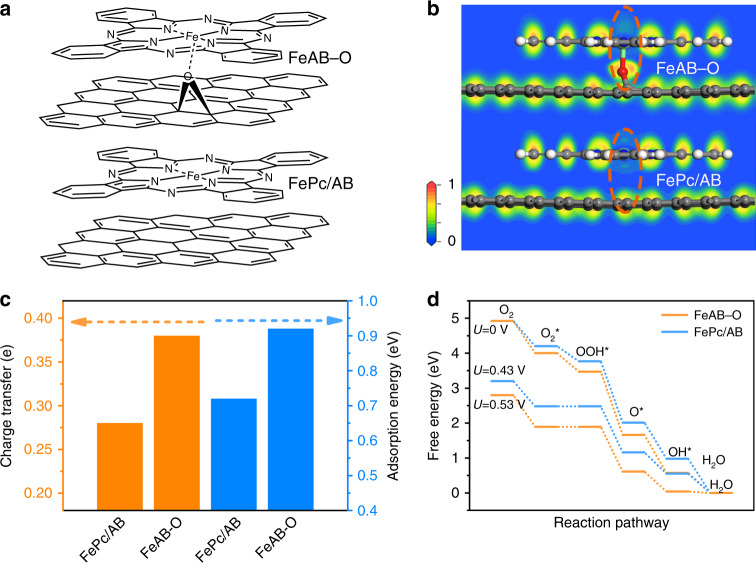
Theoretical calculations. **a** Molecular structure models. **b** Electron localization functions and **c** Bader charge transfers and the O_2_ adsorption energies of FeAB–O and FePc/AB. **d** Free energy diagrams of ORR pathways on FeAB–O and FePc/AB.

Axial O coordination in FeAB–O and no O coordination in FePc/AB were clearly showed in the schematic diagrams (Fig. 1a), which leads to obvious differences on the electron localization functions (Fig. 1b). As expected, the FePc/AB shows a symmetric charge distribution. Instead, strong electronic localization on the axial O atom accompanying with axial asymmetrical electronic distribution of O–FeN_4_ sites can be observed in FeAB–O. By analyzing the charge density differences and spin density (Supplementary Figs. 1 and 2), we found that the charges number and spin polarization of the symmetrical FeN_4_ site did not significantly change, due to their weaker interaction. However, the axial O coordination accepts partial charges from the FeN_4_ site to form the electron localization in FeAB–O^28–30^, which break the symmetry of electronic density near the FeN_4_ site and change the spin polarization of FeN_4_ site, obviously.

To study the interaction between catalysts and the adsorbed O_2_, the O_2_ adsorption energy, charge density differences (between catalysts and adsorbed O_2_), Bader charge analysis, projected density of states (PDOS), and spin density were performed (Fig. 1c, Supplementary Figs. 3 and 4)^31^. As predicted, the FeAB–O shows much higher O_2_ adsorption energy of 0.92 eV than that of FePc/AB with 0.72 eV. Correspondingly, the charge transfers from FeAB–O and FePc/AB to the adsorbed O_2_ (Fig. 1c) are 0.38 and 0.28 *e*, respectively. In addition, the PDOS and spin density of O_2_* adsorption on the FeAB–O show that the 3*d* electrons of Fe and the 2*p* electrons of O form stronger hybrid states below the Fermi level, and the spin polarization of oxygen molecule was broken (Supplementary Figs. 3 and 4). These results reveal that electronic localization on axial O coordination (O–FeN_4_ sites) enhances O_2_ adsorption and activation.

To study the effect of electronic localization on the ORR processes, the free energies of ORR pathways on FeAB–O and FePc/AB were calculated (Fig. 1d). The free energy diagrams also show that the intermediate species adsorbed on FeAB–O is more stable than that on Fe/AB. Both the rate-determining steps on FeAB–O and FePc/AB are the oxygen adsorption/activation steps:

1 $${\mathrm{O}}_2 \ast + {\mathrm{H}}^ + + e^ - \to {\mathrm{OOH}} \ast$$

Thus, the stable adsorption of reactant (oxygen) can facilitate the process of ORR. The corresponding overpotential of ORR on FeAB–O and FePc/AB are 0.70 and 0.80 V, respectively, suggesting the superior ORR performance of FeAB–O than that of FePc/AB. Therefore, the axial Fe–O coordination-induced electronic localization to improve the O_2_ adsorption and activation can boost the ORR performance.

### Catalyst synthesis and characterization

**Fig. 2:**
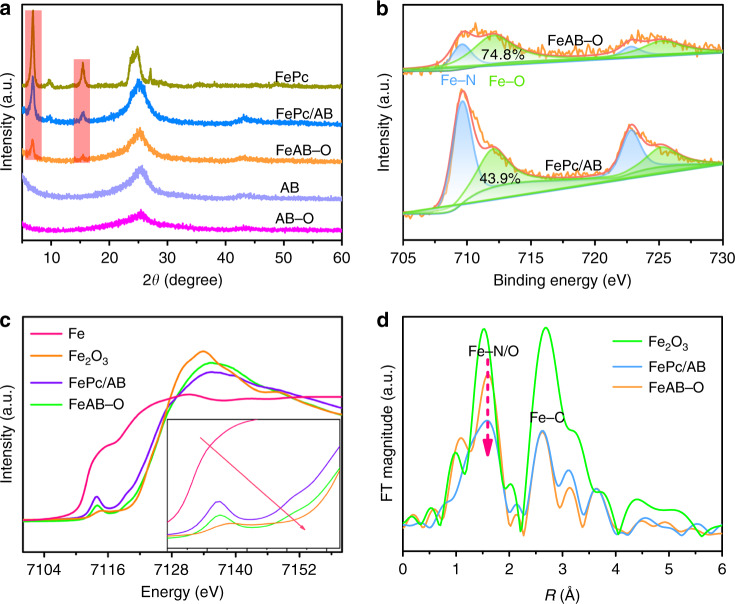
Structure characterization. **a** XRD patterns of the pristine FeAB–O, FePc/AB, AB, AB–O, and FePc. **b** XPS Fe 2*p* spectra of the FeAB–O and FePc/AB. **c** XANES spectra at Fe K-edge of the FeAB–O, FePc/AB, Fe, and Fe_2_O_3_. **d** Extended X-ray absorption fine structure (EXAFS) spectra of Fe K-edge in the FeAB–O, FePc/AB, and Fe_2_O_3_.

Inspired by the theoretical prediction, the FeAB–O catalyst was prepared by compositing of FePc with the AB–O in dimethyformamide (DMF) solution. The control sample without O coordination (FePc/AB) was obtained by direct physical mixture of FePc and AB. X-ray diffraction (XRD) patterns and Fourier transform infrared (FT-IR) spectra reveal that both FeAB–O and FePc/AB are comprised of carbon and FePc (Fig. 2a and Supplementary Fig. 5)^32,33^. Scanning electron microscope (SEM) and transmission electron microscope (TEM) images (Supplementary Figs. 6a–d and 7a) show uniform carbon nanoparticles, and there is no agglomerate FePc (Supplementary Fig. 6e) to be detected on the carbon matrix in FeAB–O. The elemental mapping from Supplementary Fig. 7b–f display overlapped distribution of C, N, O, and Fe, verifying the uniform distribution of FePc in FeAB–O.

In order to investigate the presence of axial O coordination, high-resolution X-ray photon spectroscopy (XPS) spectra and synchrotron X-ray absorption spectra were conducted (Fig. 2b–d). The fitted ratio of Fe–O to Fe–N bonds in FeAB–O shows obvious increase compared with that in FePc/AB^34^, indicating more axial O coordination with FeN_4_ sites in FeAB–O. X-ray absorption near-edge spectra (XANES) of Fe K-edge (Fig. 2c) show obvious positive shift in FeAB–O compared with in FePc/AB, indicating the change of electronic structure of Fe^35^. Moreover, a pre-edge peak around 7114 eV can be indexed to the square-planar and centrosymmetric Fe–N_4_ structure of FePc^36–38^. It should be mentioned that FeAB–O exhibits a lower peak intensity of in-plane FeN_4_ structure than that of FePc/AB, which can be attributed to the axial coordination breaking the in-plane Fe–N_4_ structure^17,39^. Additionally, extended X-ray absorption fine structure (EXAFS) spectra of Fe K-edge show that the coordination number of Fe in FeAB–O is higher than the precise four nitrogen-coordination (FeN_4_) and lower than the six-oxygen coordination (FeO_6_) in Fe_2_O_3_, respectively^40,41^. These characterization results prove the formation of axial O coordination between the FePc and oxygen group of AB–O in FeAB-O.

**Fig. 3:**
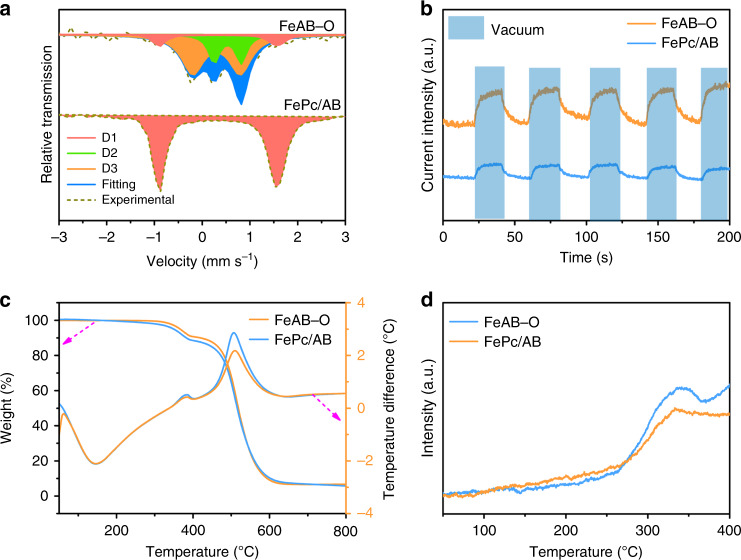
The Oxygen adsorption ability. **a** ^57^Fe Mössbauer transmission spectrum. **b** The oxygen adsorption–desorption tests. **c** TGA curves in air atmosphere. **d** The O_2_-TPD curves of FeAB–O and FePc/AB.

To obtain more structure information, the ^57^Fe Mössbauer spectra were collected at 300 K. As shown in Fig. 3a, the Mössbauer spectrum of FePc/AB has a doublet peaks (D1), which can be assigned to the square planar FeN_4_ species^20^. As for FeAB–O, there is a small D1 doublet peaks and two obvious D2 and D3 doublet peaks. The D2 peaks are from the O–FeN_4_ species, and the D3 peaks can be attributed to the O–FeN_4_ sites with surface-adsorbed O_2_ molecule (O–FeN_4_–O_2_)^39^. No clear O_2_ adsorption signal can be observed in FePc/AB. These results confirmed the axial O coordination of O–FeN_4_ and the enhanced O_2_ adsorption.

To prove the enhanced O_2_ adsorption, the O_2_ adsorption–desorption performances were measured (Fig. 3b–d and Supplementary Fig. 8). Obviously, FeAB–O exhibits stronger O_2_ adsorption response than FePc/AB, suggesting better O_2_ adsorption ability of FeAB–O than that of FePc/AB^42^. The O_2_ temperature-programmed desorption (TPD) measurements were also performed to investigate the O_2_ adsorption property. According to the thermogravimetry analysis (TGA) of FeAB–O and FePc/AB (Fig. 3c), the weight loss at 380 and 507 °C can be ascribed to the decomposition of FePc and carbon, respectively. In Fig. 3d, the O_2_-desorption peaks located at 340 °C can be assigned to the release of chemistry-adsorbed O_2_ in the samples^43^. Interestingly, the O_2_-desorption peak of FeAB–O is higher than that of FePc/AB, indicating the robust O_2_ adsorption ability of FeAB–O.

### Evaluating catalyst performance for ORR

**Fig. 4:**
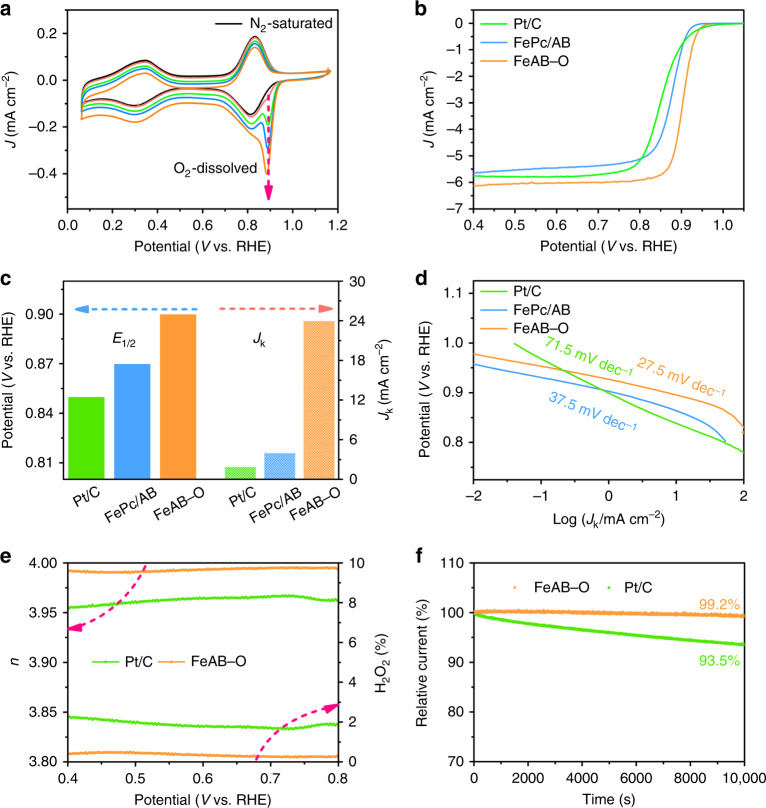
Electrochemical ORR performances. **a** Cyclic voltammetry profiles of FeAB–O in N_2_-saturated and O_2_-dissolved 0.1 M KOH solution. **b** ORR polarization curves of FeAB–O, FePc/AB, and Pt/C in O_2_-saturated 0.1 M KOH. **c** Values of half-wave potentials and *J*_k_ at 0.88 V of FeAB–O, FePc/AB, and Pt/C. **d** Corresponding Tafel plots of FeAB–O, FePc/AB, and Pt/C. **e** Electron transfer numbers and proportion of produced H_2_O_2_ in FeAB–O and Pt/C. **f** I–t chronoamperometry responses (in O_2_-saturated 0.1 M KOH with a rotation of 1600 rpm) of FeAB–O and Pt/C.

To identify the electrochemical ORR properties of catalysts, the cyclic voltammetry (CV) was measured in 0.1 M KOH. As presented in Fig. 4a, the CV curve of FeAB–O in N_2_-saturated electrolyte contains two pairs of peaks located at 0.8 and 0.3 V. The former is indexed to the reduction/oxidation peaks of Fe^3+^/Fe^2+^, the latter is signed to redox couple of Fe^2+^/Fe^+^ ^38^. With the increasing of dissolved O_2_ molecule, obvious new reduction peaks located at about 0.9 V occur and increase, even beyond the reduction peak of Fe^3+^/Fe^2+^, suggesting the active site of variable O–FeN_4_. However, the FePc/AB and the pristine FePc display was negligible in these two groups of peaks in Supplementary Fig. 9a, suggesting that the oxygenic carbon coordination with FePc via Fe–O is beneficial for electronic delocalization of Fe to form the active site. The CV curves of the control samples under N_2_ and O_2_-saturated electrolyte were also conducted and shown in Supplementary Fig. 9b–d. All the samples exhibit obvious oxygen reduction peaks and FeAB–O displays the most positive potential, indicating the optimal ORR performance of FeAB–O.

Next, the linear scan voltammetry (LSV) curves of FeAB–O and FePc/AB were conducted to further study their ORR properties (Fig. 4b). The theoretical calculations, electrochemical impedance spectroscopy (EIS), and resistance tests (Supplementary Fig. 10 and Supplementary Note 1) demonstrate that the electrons can transfer from the electrode to FePc molecule through the Fe–O bonds with the help of electric field. Thus, FeAB–O presents a remarkable *E*_1/2_ of 0.90 V and a calculated kinetic current density (*J*_k_) of 24.0 mA cm^−2^ at 0.88 V, which are much superior to FePc/AB (*E*_1/2_ = 0.87 V, *J*_k_ = 1.9 mA cm^−2^ at 0.88 V) and Pt/C (*E*_1/2_ = 0.85 V, *J*_k_ = 4.0 mA cm^−2^ at 0.88 V). Instead, the AB, AB–O, and FePc only exhibit inferior half-wave potentials (*E*_1/2_) and limited current density (*J*_L_) in Supplementary Fig. 11a. Moreover, FePc is physically mixed with AB–O (FePc/AB–O) to exclude the effect of carbon substrate. As expected, the FePc/AB–O displays the alike performance of FePc/AB in Supplementary Fig. 11b. Based on these results, the introduction of axial O coordination in O–FeN_4_ sites can greatly boost the performance of ORR. The ORR catalytic activity of FeAB–O is superior to most of reported Fe–N–C catalysts in recent literatures (Supplementary Table 1). Notably, the FeAB–O has the most excellent Tafel slope of 27.5 mV dec^−1^, which is lower than those of FePc/AB (37.5 mV dec^−1^) and Pt/C (71 mV dec^−1^), confirming the fastest kinetic process of FeAB–O for O_2_ adsorption/activation and ORR.

The selectivity of ORR in FeAB–O was studied by rotating ring disk electrode (RRDE) measurements. Compared with Pt/C, the higher electron transfer number and lower H_2_O_2_ yield can be observed in FeAB–O (Fig. 4e), indicating the ORR on FeAB–O is a typical four-electron reduction process, and the main product is H_2_O.

To explore the practical application of FeAB–O, long-term catalytic stability and methanol tolerance tests were performed at reduced potential of 0.4 V vs. RHE. As for the traditional Pt/C catalyst, Pt nanoparticles tend to aggregate after long-term ORR measurements, which leads to a decrease in activity and durability^44^. In this work, the ORR current density of FeAB–O maintained a level of 99.2% for over 10,000 s chronoamperometric I-t tests, exceeding that of Pt/C (93.5%) and FePc/AB (88.3%) (Fig. 4f and Supplementary Fig. 12a). The outstanding durability of FeAB–O for ORR can be attributed to the high dispersity and stability of the O–FeN_4_ sites^17,45^. No current oscillation was observed in FeAB–O when methanol is added (Supplementary Fig. 12b), while a clear decline of current appears in Pt/C. A home-made aluminum–air battery was used to evaluate the practical performance of FeAB–O. The battery with FeAB–O as cathode catalyst shows higher open potential than that with Pt/C as cathode catalyst (Supplementary Fig. 12c). The corresponding discharge plots of long-term discharge were performed at current density of 50 mA cm^−2^. As shown in Supplementary Fig. 12d, the FeAB–O exhibits superior performance than that of the commercialized Pt/C. These results demonstrated that FeAB–O has excellent potential for practical application.

To further confirm the importance of the axial O coordination of O–FeN_4_, FeAB with less axial O coordination was prepared by compositing of FePc with AB treated by O_2_-plasma for only 10 min in DMF solution. XRD and FT-IR characterization results (Supplementary Fig. 13a, b) prove the presence of FePc and carbon in FeAB. The Fe2*p* XPS spectrum (Supplementary Fig. 13c) indicates the presence of axial O coordination in FeAB. The EXAFS results (Supplementary Fig. 13d) show the order of Fe coordination number is FeAB–O > FeAB > FePc/AB. These structural characterizations confirm the axial O coordination in FeAB is between FeAB–O and FePc/AB. As we expected, the electrochemical ORR performance of FeAB (Supplementary Fig. 14a, b) is also between FeAB–O and FePc/AB, confirming the axial O coordination induced the electronic localization, which improves the O_2_ adsorption and then boosts ORR activity of catalysts.

## Discussion

In summary, we proposed a coordination-induced electronic localization strategy to tune the O_2_ adsorption ability and ORR performance of FeN_4_ sites in FePc. DFT calculations demonstrated that the axial O coordination of O–FeN_4_ sites breaks the symmetrical electronic density and promotes the electronic localization of Fe sites. XPS, XAS, Mössbauer spectra, and O_2_ adsorption/desorption processes indicated the enhanced ORR catalytic activity is ascribed to the strengthened O_2_ adsorption and accelerated charge transfer from Fe to O_2_ molecule. As a result, the FeAB–O with optimal axial O coordination exhibited a record Tafel slope of 27.5 mV dec^−1^ and one of the best half-wave potential of 0.90 V vs. RHE, which was much superior to commercial Pt/C. The axial O coordination number is positively correlated to ORR performance. This work provides a new strategy to regulate the electronic localization property of catalytic active sites for affecting the adsorption of the reactants and accelerating catalytic reactions.

## Methods

### Chemicals and materials

FePc, dimethylformamide (DMF), potassium chloride (KCl), indium hydroxide (In(OH)_3_), zinc oxide (ZnO), sodium stannate (Na_2_SnO_3_), and potassium hydroxide (KOH) were bought from Shanghai Aladdin reagent co. Ltd. Pt/C (20 wt%) and the raw AB were purchased from Alfa Aesar and Shenzhen Kejing co. Ltd, respectively. All of the chemical reagents were used as received without any other purification.

### Synthesis of catalysts

The surface of the AB was decorated with oxygen-containing groups by O_2_-plasma treatment. Typically, the raw AB was treated in the O_2_ plasma for 30 min with 100 W generator power (denoted as AB–O). Then, the as obtained AB–O (25 mg) was added into 60 mL of DMF solution encompassed 5 mg of FePc. To get the uniform suspension, the mixture solution was subjected to ultrasonical treatment for 1 h and then stirred overnight at room temperature. Finally, the FeAB–O composite was collected by filtration of the resulting solution and washing with ethanol. The obtained sample was dried in vacuum at 60 °C for 12 h. The FeAB with less axial O coordination was obtained by the same steps just by replacing the AB–O matrix with AB treated by O_2_-plasma for 10 min at 100 W. The FePc/AB and FePc/AB–O were obtained by direct physical mixture of FePc with the AB and AB–O, respectively.

### Characterizations

XRD data was collected by using a RIGAKU Rint-2000 X-ray diffractometer (graphite monochromatized Cu-Kα radiation with *λ* = 1.54184 Å). X-ray photoelectron spectroscopy (XPS) was measured by Thermo ESCALAB 250XI. FTIR measurements were performed by the Thermo iS50. The thermogravimetric experiments were conducted on TG 209 F3 Tarsus under the air atmosphere from the room temperature to 900 °C with heating rate of 10 °C min^−1^. Scanning electron microscopy (SEM) was measured by a Quanta 200 field-emission SEM system. The transmission electron microscopy (TEM) images were achieved on Tecnai G2 F20. The ^57^Fe Mössbauer spectra were achieved by using an MS-500 instrument (Germany, Wissel) in transmission geometry with constant acceleration mode at room temperature. The O_2_-TPD of the samples was measured using AutoChem II 2920 apparatus. The catalyst (100 mg) was pretreated at 150 °C and purged with helium (He) for 2 h, and then cooled down to room temperature. And then, the catalyst was purged with 5% O_2_/He at 25 °C for 2 h. Finally, the desorption profile of O_2_ was recorded online under the atmosphere of He.

### Electrochemical measurements

All of the electrochemical experiments were implemented with an electrochemical station of Auto Lab in a typical three-electrode system. The Ag/AgCl (saturated KCl) electrode, carbon rod, and glassy carbon electrode (GCE) were used as the reference electrode, counter electrode, and working electrode, respectively. In this work, all electrode potentials were referenced to the reversible hydrogen elecrtrode (RHE) based on the following calculation equations:

2 $$E_{{\mathrm{RHE}}} = E_{{\mathrm{AgCl}}}^0 + E_{{\mathrm{AgCl}}} + 0.059 \times {\mathrm{pH}}$$

where $E_{{\mathrm{AgCl}}}^0$ (saturated KCl) = 0.197 V (25 °C).

The catalyst ink was prepared by ultrasonic dispersion of 4 mg of catalyst in a hybrid solution included 60 µL of Nafion (5 wt%), 470 µL of alcohol, and 470 µL of H_2_O. All of the catalysts were cast onto the RDE (0.19625 cm^−2^) and RRDE (0.2475 cm^−2^) with a loading amount of 0.2 mg cm^−2^, and contrast sample of Pt/C was dropped RRDE with a loading amount of 0.1 mg cm^−2^. Cyclic voltammograms (CV) measurements were performed with scan rate of 50 mV s^−1^ in the N_2_ or O_2_-satureated 0.1 M KOH solution. The catalytic activity of samples was evaluated by using linear sweep voltammetry (LSV) at scan rate of 10 mV s^−1^ with different rotation rates. The electron transfer number (*n*) of catalysts was calculated through the Koutecky–Levich (K–L) equations:

3 $$\frac{1}{J} = \frac{1}{{J_{\mathrm{L}}}} + \frac{1}{{J_{\mathrm{{k}}}}} = \frac{1}{{B\omega ^{1/2}}} + \frac{1}{{J_{\mathrm{{k}}}}}$$

4 $$B = 0.62nFD_{\mathrm{{o}}}^{2/3}{\nu}^{ - 1/6}C_{\mathrm{{o}}}$$

where *J*, *J*_L_, and *J*_k_ represents the measured, diffusion-limiting, and the kinetic current density, individually. *ω* is the electrode-rotating angular velocity, *F* is the Faraday constant (96,485 C mol^−1^), *D*_o_ is the diffusion coefficient of O_2_ (1.9 × 10^−5^ cm^2^ s^−1^ in 0.1 M KOH), *ν* is kinetic viscosity (0.01 cm^2^ s^−1^) of the electrolyte, and *C*_o_ is the density of O_2_ (1.2 × 10^−6^ mol cm^−3^).

Tafel slope was achieved from the Tafel equation:

5 $$E = {a} + {b}\,{\mathrm{log}}\left( {J_k} \right)$$

where *E* is the applied potential of LSV tests, *a* is a constant, *b* is the Tafel slope and *J*_k_ is the kinetic current density. Moreover, the yields of peroxide species and the electron transfer number can be calculated from the LSV of RRDE measurement at 1600 rpm via as following equation:

6 $${n} = 4\frac{{I_{\mathrm{{D}}}}}{{I_{\mathrm{{D}}} + I_{\mathrm{{R}}}/N}}$$

7 $${\mathrm{{H}}}_2{\mathrm{{O}}}_2({\mathrm{\% }}) = 200\frac{{I_{\mathrm{{R}}}/N}}{{I_{\mathrm{{D}}} + \left( {I_{\mathrm{{R}}}/N} \right)}}$$

where *I*_D_ and *I*_R_ is the disk current and ring current, respectively. The *N* represents the current collection efficiency equaled to 0.37 of the RRDE in our experimental system.

### Assembly of Al–air batteries

In a typical Al–air batteries, the polished aluminum plate is used as anode. The electrolyte is 6 M KOH contained 0.0005 M In(OH)_3_, 0.0075 M ZnO, and 0.01 M Na_2_SnO_3_; the gas diffusion electrode with catalyst-loading amount of 2.0 mg cm^−2^ is employed as cathode in a home-made cell model. As a control, the commercial Pt/C (20 wt%) was also assembled in similar mode. The measurement of batteries was performed on the LAND testing system.

### Computation methods

Our simulation study was calculated by using the Vienna ab initio simulation package (VASP)^46^. The PAW potentials describe the interaction of electron–ion^47^. The generalized gradient approximation of Perdew–Burke–Ernzerhof (PBE) was employed to describe the electron–electron exchange and correlation functional^48^. A plane wave cutoff energy of 400 eV was applied in our calculations. A rectangular supercell containing 180 carbon atoms are used as substrate. Spin-polarized calculations were employed for all systems. van der Waals (VDW) forces were corrected with the D2 method of Grimme^49^. The Gamma-point-only grid was used during the optimization. The convergence criterion was set 0.02 eV Å^−1^ for the force and 10^−5^ eV per atom for energy. We used the correlation energy (*U*) of 4 eV and the exchange energy (*J*) of 1 eV for Fe 3*d* orbitals^50^.

The Gibbs free energy can be expressed as

8 $${\Delta}G = {\Delta}E + {\Delta}{\mathrm{ZPE}} - T \cdot {\Delta}S$$

where Δ*E* is the reaction energy calculated by the DFT methods, ΔZPE the changes in zero-point energies, and Δ*S* the entropy during the reaction, respectively.

## Supplementary information

Supplementary Information

## Data Availability

The data that support the findings of this study are available from the corresponding author on reasonable request.
