# Supplementary material for: Iron phthalocyanine with coordination induced electronic localization to boost oxygen reduction reaction
Source: Nat Commun. 2020 Aug 20;11:4173. doi: 10.1038/s41467-020-18062-y (PMC7441147; doi:10.1038/s41467-020-18062-y)
Supplement: Supplementary file 1 — Supplementary Information [file 41467_2020_18062_MOESM1_ESM.pdf]

Supplementary Information

# Iron phthalocyanine with coordination induced electronic localization to boost oxygen reduction reaction

Chen *et al*.

## Supplementary Figures

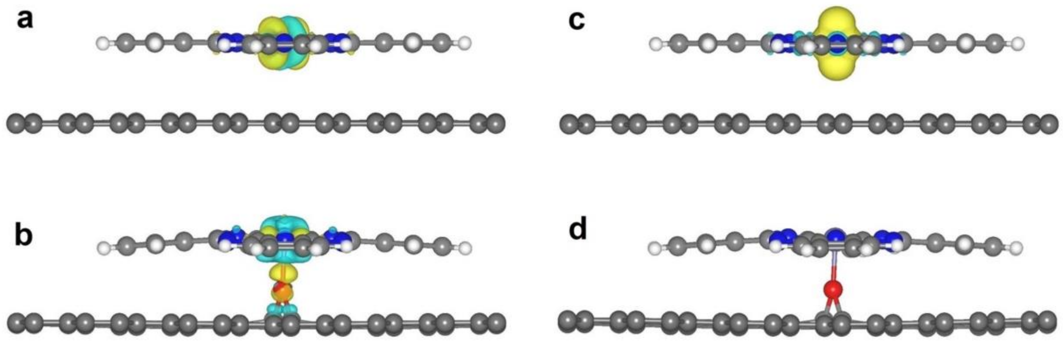


**Supplementary Figure 1. The charge density differences and spin density.** The charge density differences of **a** FePc/AB and **b** FeAB-O. The isosurface level set to 0.003 e Å$^{-3}$, yellow stands for positive area and gray stands for negative area. The spin density of **c** FePc/AB and **d** FeAB-O (Yellow stands for spin up and gray stands for spin down).

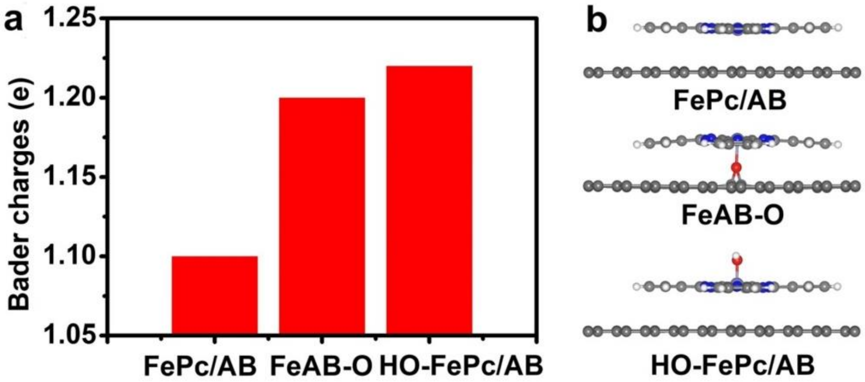


**Supplementary Figure 2. The number of Bader charges transfer and configurations.** **a** The number of Bader charges transfer at Fe sites in FePc/AB, FeAB-O and HO-FePc/AB. **b** The configurations of FePc/AB, FeAB-O and HO-FePc/AB.

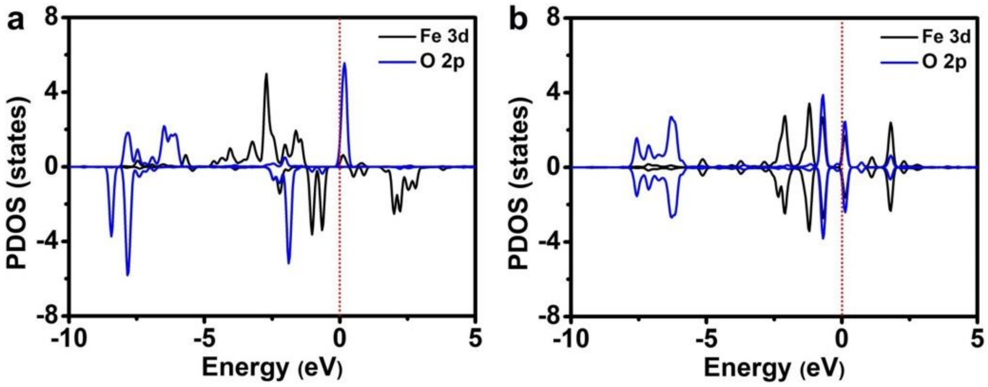


**Supplementary Figure 3. The projected density of states.** The projected density of states of $O_2$* adsorption on **a** FePc/AB and **b** FeAB-O.

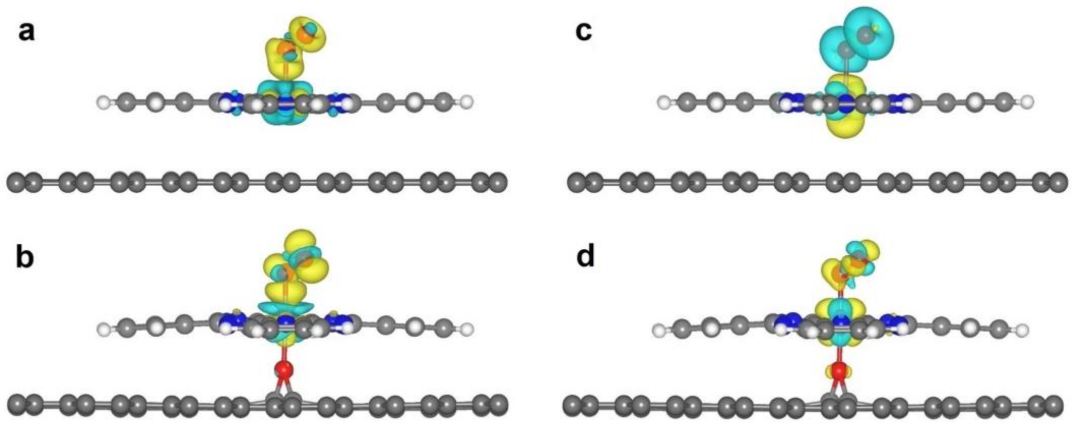


**Supplementary Figure 4. The charge density differences and spin density.** The charge density differences of the $O_2$ adsorption on **a** FePc/AB and **b** FeAB-O. The isosurface level set to 0.003 e $Å^{-3}$, yellow stands for positive area and gray stands for negative area. The spin density of $O_2$ adsorption on **a** FePc/AB and **b** FeAB-O (Yellow stands for spin up and gray stands for spin down).

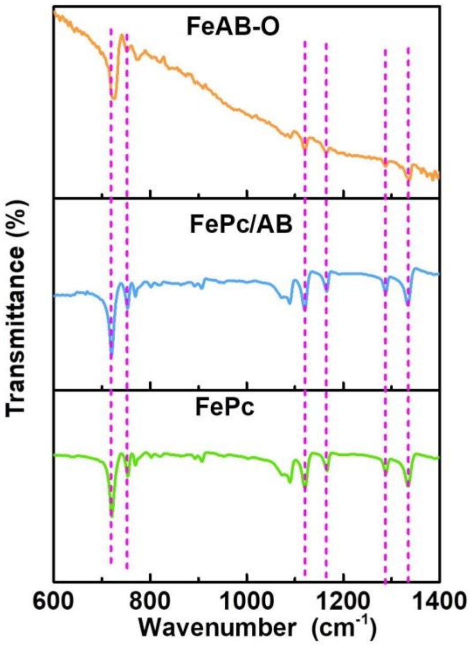


**Supplementary Figure 5. FTIR characterizations.** FTIR spectra of FeAB-O, FePc/AB and FePc.

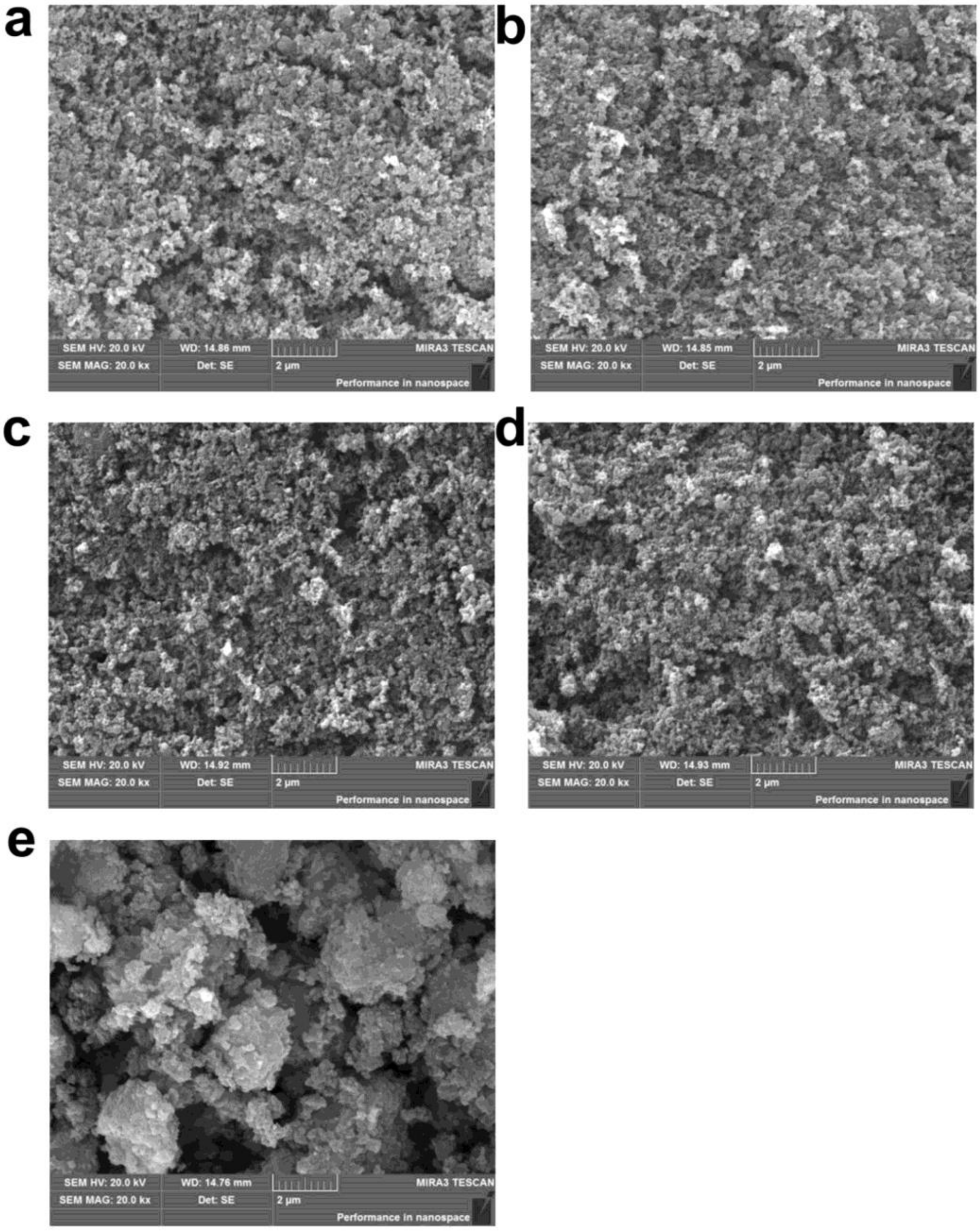


**Supplementary Figure 6. SEM characterizations.** SEM images of **a** AB, **b** AB-O, **c** FePc/AB, **d** FeAB-O and **e** FePc.

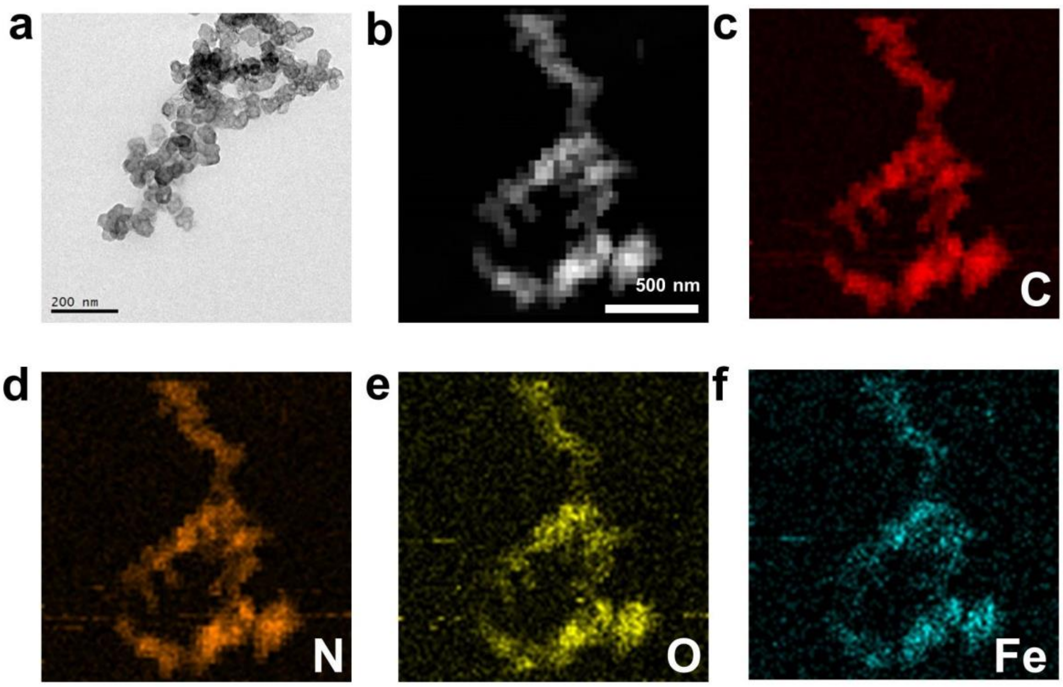


**Supplementary Figure 7. TEM characterizations. a** TEM images of FeAB-O. **b** A typical TEM image of FeAB-O for elemental distribution and the elemental mapping of **c** carbon, **d** nitrogen, **e** oxygen, **f** iron.

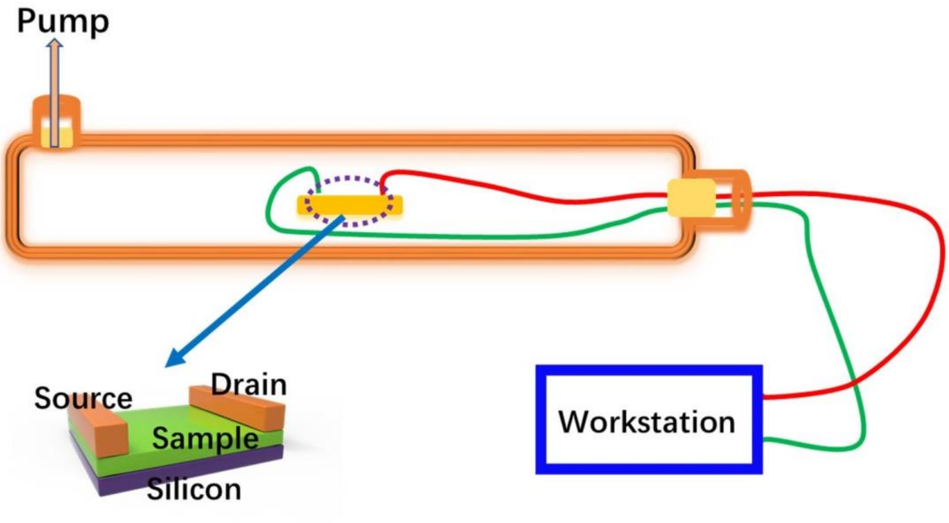


**Supplementary Figure 8. Schematic illustration of $O_2$ adsorption-desorption measurements.**

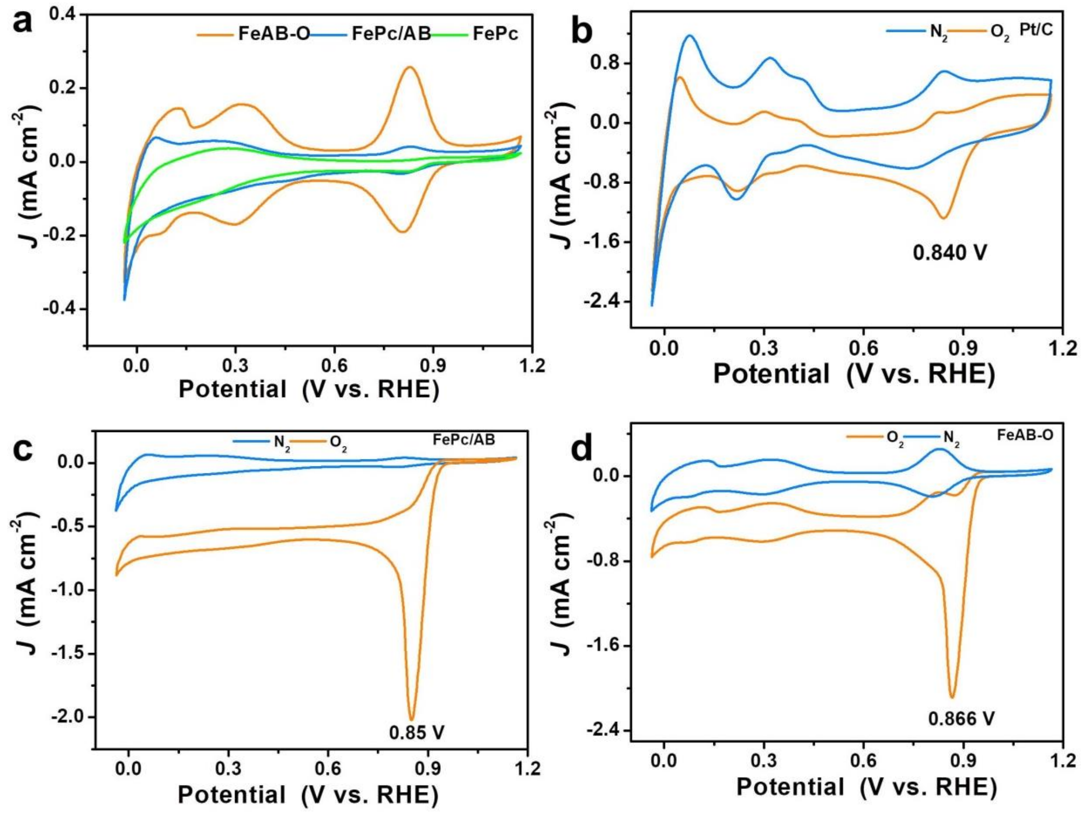


**Supplementary Figure 9. CV measurements. a** CV profiles of FePc, FePc/AB and FeAB-O in $N_2$-saturated 0.1 M KOH solution. **b** CV profiles of Pt/C, **c** FePc/AB and **d** FeAB-O in $N_2$- and $O_2$-saturated 0.1 M KOH solution.

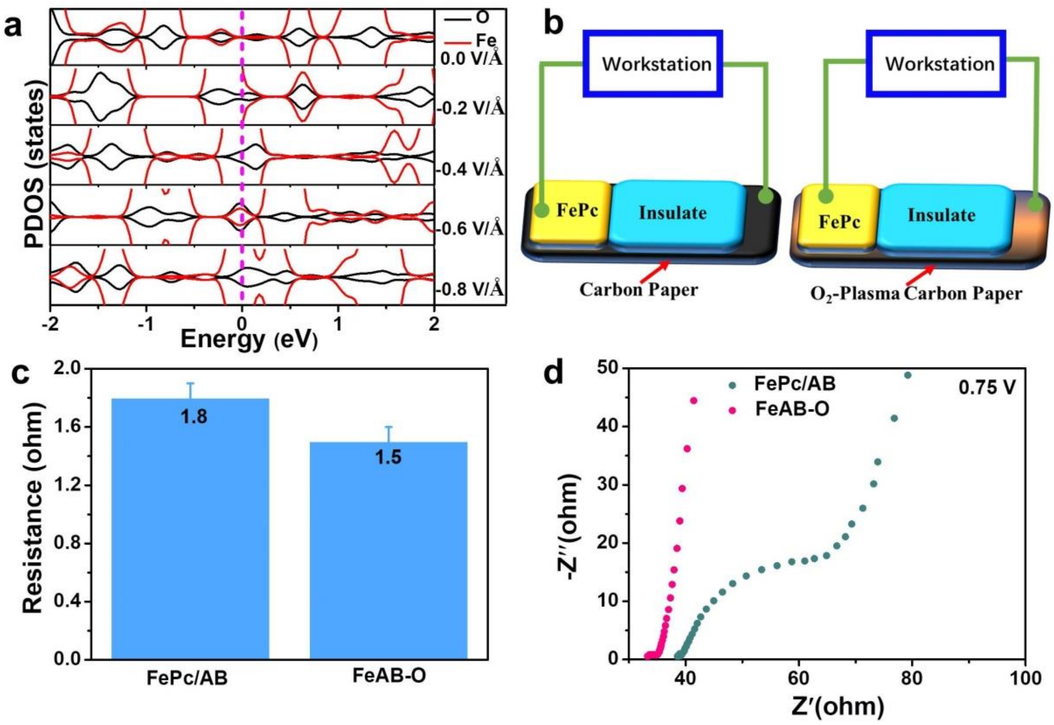


**Supplementary Figure 10. Electrical conductivity of Fe–O bond. a** The projected density of states (PDOS) of the Fe and O on the FeAB-O under an electric field (ε) from 0 to –0.8 V/Å. **b** Schematic illustration of resistance tests. **c** The resistance tests results of FePc/AB and FeAB-O. **d** The EIS of FePc/AB and FeAB-O at 0.75 V in $O_2$-saturated 0.1 M KOH solution.

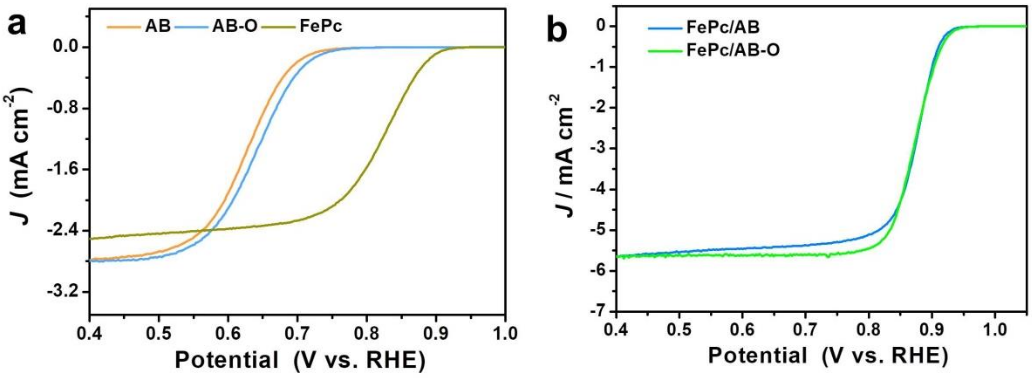


**Supplementary Figure 11. ORR polarization curves. a** LSV of AB, AB-O and FePc in $O_2$-saturated 0.1 M KOH solution. **b** LSV of FePc/AB and FePc/AB-O in $O_2$-saturated 0.1 M KOH solution.

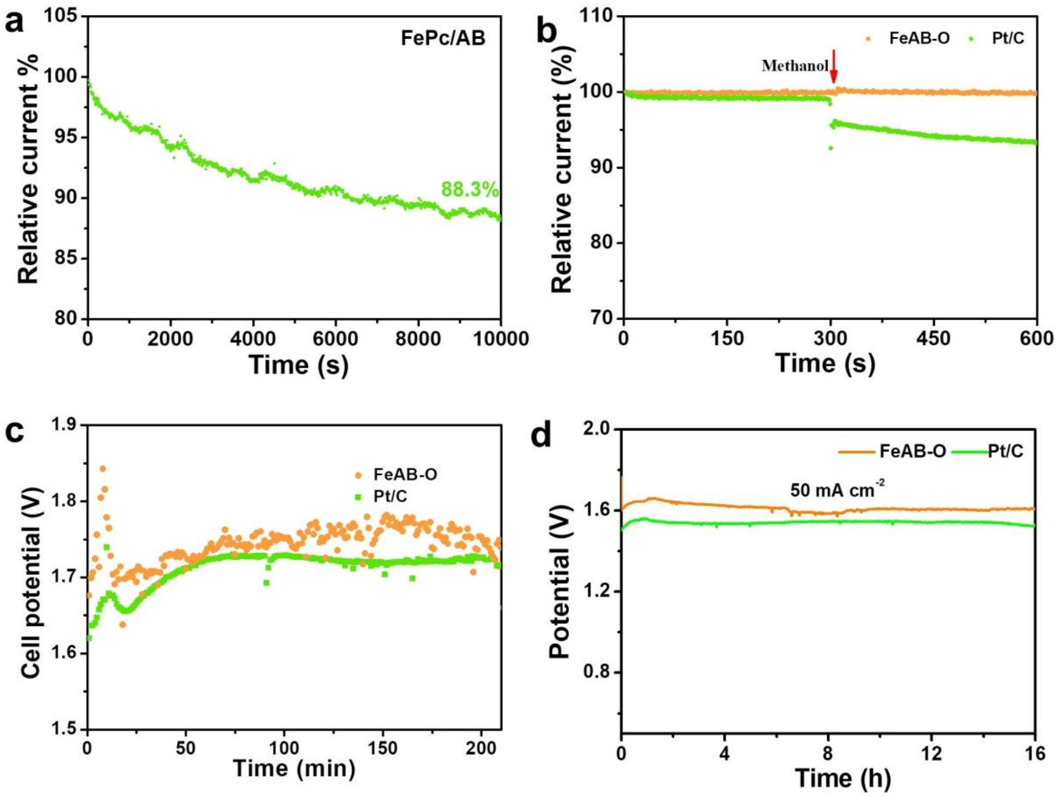


**Supplementary Figure 12. Electrochemistry performances of catalysts. a** I-t chronoamperometry responses of FePc/AB in $O_2$-saturated 0.1 M KOH with a rotation of 1600 rpm. **b** Methanol tolerance tests of FeAB-O and Pt/C. **c** Open circuit potential-time curve of the home-made Al-Air battery. **d** Long-time discharge curves at discharge density of 50 mA $cm^{-2}$.

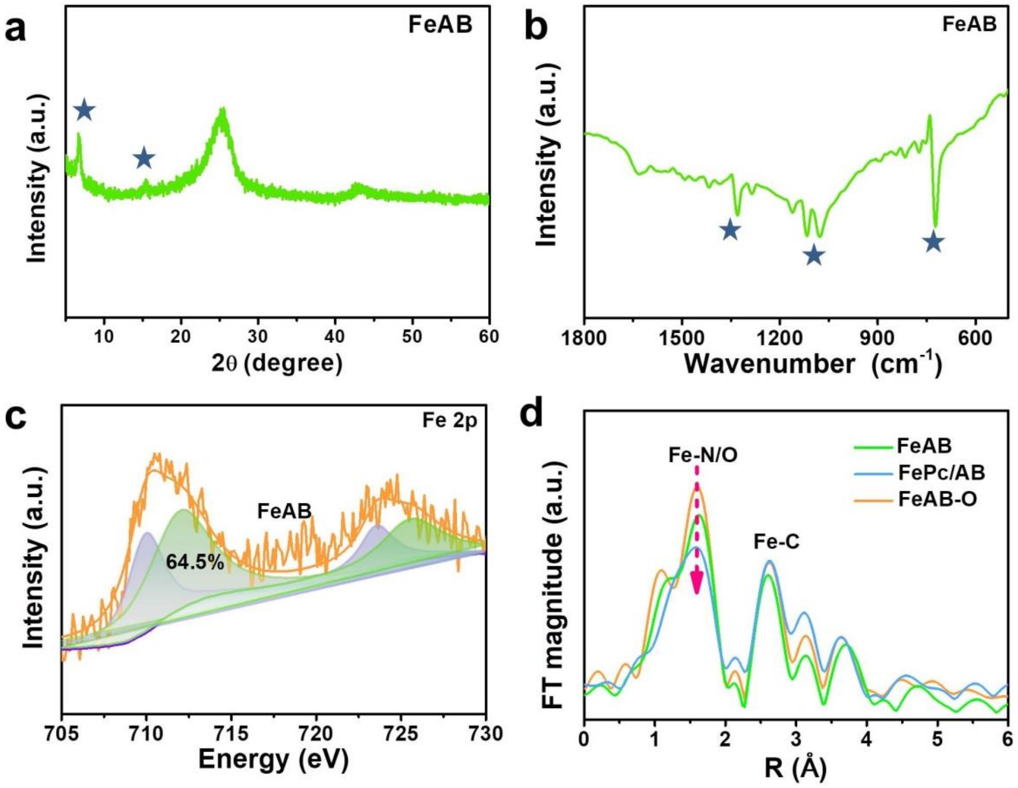


**Supplementary Figure 13. Materials characterizations of FeAB. a** XRD pattern. **b** FTIR spectrum. **c** Fe 2p XPS spectrum. **d** Fourier transforms of Fe K-edge spectra of FeAB, FePc/AB and FeAB-O.

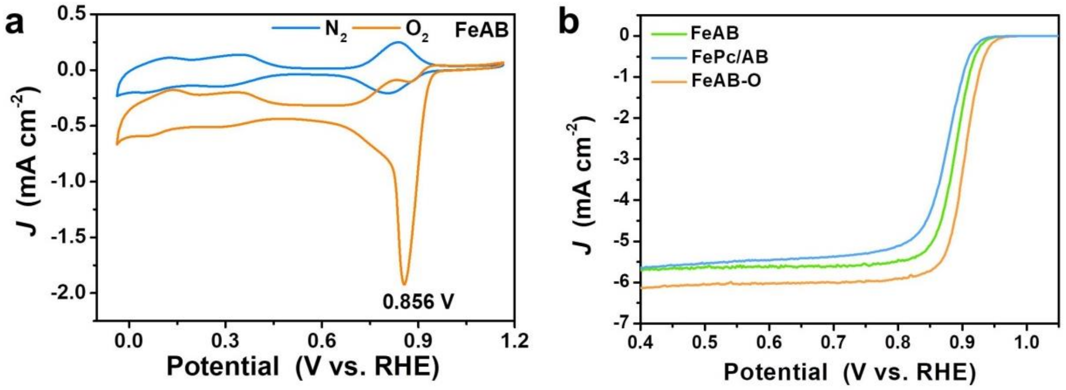


**Supplementary Figure 14. CV and LSV in 0.1 M KOH solution. a** Cyclic voltammetry profiles of FeAB in $N_2$- and $O_2$-saturated 0.1 M KOH solution. **b** ORR polarization curves of FePc/AB, FeAB and FeAB-O in $O_2$-saturated 0.1 M KOH solution.

## Supplementary Tables

**Supplementary Table 1.** Comparison of ORR performance in the alkaline media between prepared catalysts and the reported Fe–N–C catalysts in literatures.

| Electrocatalysts | $E_{onset}$(V) | $E_{1/2}$(V) | Tafel (mV $dec^{-1}$) | Reference |
|---|---|---|---|---|
| FeAB-O | 0.98 | 0.90 | 27.5 | This work |
| FePc/AB | 0.96 | 0.87 | 37.5 | This work |
| Pt/C | 1.03 | 0.85 | 71 | This work |
| Fe-SAs/NSC | 1.00 | 0.87 | - | 1 |
| Fe-N/P-C-700 | 0.94 | 0.87 | - | 2 |
| Fe/OES | 1.00 | 0.85 | - | 3 |
| PCNT@Fe@GL | 0.97 | 0.87 | 61.9 | 4 |
| Fe/SNC | 0.97 | 0.85 | - | 5 |
| FePhen@MOFArNH$_3$ | 1.03 | 0.86 | - | 6 |
| $Fe_2$-$Z_8$-C | 0.98 | 0.87 | - | 7 |
| pfSAC | 1.03 | 0.91 | 31.7 | 8 |
| FeSAs/PTF-600 | 1.01 | 0.87 | - | 9 |
| SA-Fe-HPC | 1.0 | 0.89 | 49 | 10 |
| FePc/$Ti_3C_2T_x$ | 0.97 | 0.89 | - | 11 |

## Supplementary Notes

**Supplementary Note 1.** To demonstrate the efficient electron transfer by the axial Fe–O covalent bonds, we calculated the PDOS of Fe and O at specified electric field strengths (0, –0.2, –0.4, –0.6, and –0.8 V/Å). As shown in Supplementary Figure 10a, the PDOS of Fe and O can through the Fermi level driven by the electric field. This result indicates that the electrons can freely transfer from the carbon substrate to FePc molecule by the axial Fe–O covalent bonds. The resistance tests were performed to further confirm the conductivity of FePc/AB and FeAB-O. As shown in Supplementary Figure 10b, the FePc was dispersed in the DMF to drop onto the carbon paper (FePc/AB) and $O_2$-plasma carbon paper (FeAB-O). From Supplementary Figure 10c, the FeAB-O exhibits the lower resistance of 1.5 ohm than counterparty of FePc/AB (1.8 ohm). The EIS also indicates the FeAB-O with inferior intrinsic resistance compared with FePc/AB in Supplementary Figure 10d. These results demonstrate the electrons can transfer from the electrode to FePc molecule through the Fe–O bond with the help of electric field.

## Supplementary References


1. Zhang, J., *et al.* Tuning the Coordination Environment in Single-Atom Catalysts to Achieve Highly Efficient Oxygen Reduction Reactions. *J. Am. Chem. Soc.* **141**, 20118-20126 (2019).
2. Yuan, K., *et al.* Boosting Oxygen Reduction of Single Iron Active Sites via Geometric and Electronic Engineering: Nitrogen and Phosphorus Dual Coordination. *J. Am. Chem. Soc.* **142**, 2404-2412 (2020).
3. Hou, C.-C., *et al.* Single-Atom Iron Catalysts on Overhang-Eave Carbon Cages for High-Performance Oxygen Reduction Reaction. *Angew. Chem. Int. Ed.* **59**, 7384-7389 (2020).
4. Ahn, S.H., Yu, X. & Manthiram, A. “Wiring” Fe-Nx-Embedded Porous Carbon Framework onto 1D Nanotubes for Efficient Oxygen Reduction Reaction in Alkaline and Acidic Media. *Adv. Mater.* **29**, 1606534 (2017).
5. Chen, P., *et al.* Atomically Dispersed Iron–Nitrogen Species as Electrocatalysts for Bifunctional Oxygen Evolution and Reduction Reactions. *Angew. Chem. Int. Ed.* **56**, 610-614 (2017).
6. Strickland, K., *et al.* Highly active oxygen reduction non-platinum group metal electrocatalyst without direct metal–nitrogen coordination. *Nat. Commun.* **6**, 7343 (2015).
7. Liu, Q., Liu, X., Zheng, L. & Shui, J. The Solid-Phase Synthesis of an Fe-N-C Electrocatalyst for High-Power Proton-Exchange Membrane Fuel Cells. *Angew. Chem. Int. Ed.* **57**, 1204-1208 (2018).
8. Peng, P., *et al.* A pyrolysis-free path toward superiorly catalytic nitrogen-coordinated single atom. *Sci. Adv.* **5**, eaaw2322 (2019).
9. Yi, J., *et al.* Atomically Dispersed Iron–Nitrogen Active Sites within Porphyrinic Triazine-Based Frameworks for Oxygen Reduction Reaction in Both Alkaline and Acidic Media. *ACS Energy Lett.* **3**, 883-889 (2018).
10. Zhang, Z., Sun, J., Wang, F. & Dai, L. Efficient Oxygen Reduction Reaction (ORR) Catalysts Based on Single Iron Atoms Dispersed on a Hierarchically Structured Porous Carbon Framework. *Angew. Chem. Int. Ed.* **57**, 9038-9043 (2018).
11. Li, Z., *et al.* The Marriage of the FeN4 Moiety and MXene Boosts Oxygen Reduction Catalysis: Fe 3d Electron Delocalization Matters. *Adv. Mater.* **30**, 1803220 (2018).
